# Safety assessment of a standardized cucumber extract (Q-Actin^™^): Oral repeat-dose toxicity and mutagenicity studies

**DOI:** 10.1016/j.toxrep.2018.10.014

**Published:** 2018-10-28

**Authors:** S. Kothari, M. Saravana, S. Muthusamy, A. Mozingo, M. Soni

**Affiliations:** a Gateway Health Alliances, 4769 Mangles Blvd., Fairfield, CA 94534, USA; b Vipragen Biosciences Pvt. Ltd., 67B, Hootagalli Industrial Area, Mysore 570 018, Karnataka, India; c RNI Consulting LLC, 822 N. A1A Hwy., Ste 310, Ponte Vedra Beach, FL 32082, USA; d Soni and Associates Inc., 973 37th Place, Vero Beach, FL 32960, USA

**Keywords:** Q-Actin, Cucumber extract, Iminosugar, ido-BR1, Safety, Toxicology

## Abstract

*Cucumus sativus* (cucumber) is one of the most widely consumed fruit vegetables worldwide. Recent discovery of iminosugars in commonly consumed fruits and vegetables has promoted the interest in isolating these compounds and understanding the potential benefits to human health. The objective of the present study was to investigate the general toxicity and mutagenic effects of an aqueous extract of cucumber (Q-Actin), standardized to ≥1% (1–2%) ido-BR1 iminosugar. Single dose of Q-Actin was well tolerated without mortality at 2000 mg/kg body weight (bw) in Sprague Dawley rats. Oral (gavage) administration of Q-Actin up to 1000 mg/kg bw/day was well tolerated followed by repeated administration for a maximum period of 90 days in Sprague-Dawley rats. There were no treatment related changes in clinical observations, ophthalmic examinations, body weights and body weight gains or feed consumption, clinical chemistry and pathological changes compared to control. The mutagenicity as evaluated by Ames assay, *in vitro* chromosomal aberration test and *in vivo* micronucleus assay did not reveal any potential of Q-Actin to induce genotoxicity. The results showed that Q-Actin is well tolerated in general toxicity studies and did not induce mutagenicity. The no-observed-adverse-effect level (NOAEL) of the standardized aqueous cucumber extract (Q-Actin) is considered to be ≥1000 mg/kg bw/day, followed by repeated administration for90 days.

## 1 Introduction

The origin of cucumber (*Cucumus sativus*) is thought to be the East Indies and this fruit has been cultivated for thousands of years. Cucumber is not only a source of food but has been long used in cosmetics and traditional remedies around the world [[1], [2], [3]]. Cucumber has a history of topical use to cool and sooth the irritated skin and its use in cosmetics continues till today [2]. Many, if not most, traditional cucumber herbal therapies were water extracts (often steeped beverages) and not alcoholic extracts and therefore aqueous extracts are being increasingly studied to understand and isolate the constituents providing health benefits. The anti-inflammatory properties of cucumber has gained popularity and some recent *in vitro* [4,5] and animal model [6] experiments have explored these effects.

**Fig. 1:**
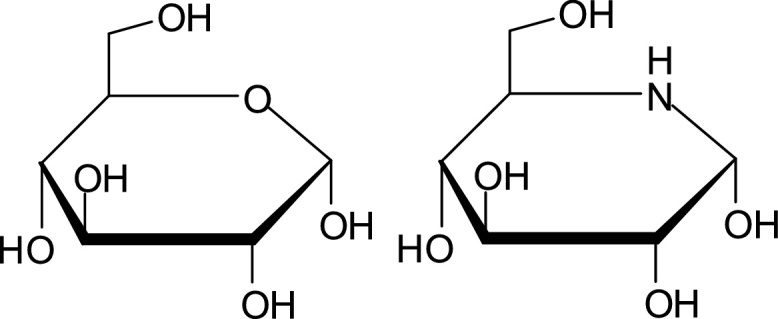
Chemical structure comparison of D-glucose and equivalent iminosugar.

Iminosugars are polyhydroxylated alkaloids that mimic the structures of monosaccharides (N. [7,8]). In the iminosugar, a nitrogen atom replaces the oxygen atom in the ring of the chemical structure of these molecules [9]. An example of D-glucose and its equivalent iminosugar is shown below in Fig. 1.

Discovery of these iminosugars is relatively recent with the first, nojirimycin (NJ) discovered in 1966 from *Streptomyces roseochromogenes*. The majority of iminosugars have been identified in the last 20 years by two research groups working together; the analysis, isolation and unequivocal identification remains difficult for most laboratories because they are hidden by small sugars and amino acids in extracts despite strong and often highly specific biological activities [3]. These naturally occurring iminosugars are classified into five structural classes: polyhdroxylated piperidines, pyrrolidines, indolizidines, pyrrolizidines and nortropanes [10]. The first iminosugars discovered were glycosidase inhibitors and the number, position and configuration of the hydroxyl groups determined the type of glycosidases which were inhibited. One example of a glycosidase-inhibiting iminosugar that is commercially available today as a dietary supplement in the United States is the piperdine 1-deoxynojirimycin (DNJ) derived from mulberry leaf.

A polyhydroxylated pipecolic acid L-*ido*-BR1 [(2*R*,3*R*,4*R*,5*S*)-3,4,5-trihydroxypiperidine-2-carboxylic acid] has been identified in an aqueous extract of cucumber manufactured under current good manufacturing practices (cGMPs) using a patented process. This proprietary extract, Q-Actin, is a 12.5:1 aqueous extract of cucumber fruit standardized to ≥1% (1–2%) *ido*-BR1. *Ido*-BR1 is a highly effective anti-inflammatory agent and is the only iminosugar found in cucumber (first reported as a natural product by Nash et al., [11]). This iminosugar, Ido-BR1, can directly reduce TNF-ɑ, a cytokine involved in acute and systemic inflammation, in human blood, leading to interest for human use. Q-Actin a standardized extract has recently been evaluated in a human clinical trial for anti-inflammatory effects with promising results [12].

Given the potential use of products containing Ido-BR1 iminosugar, it is important to make sure that the increased exposure to these sugars from products like Q-Actin is safe in humans. The object of the present study is to evaluate the preclinical safety of Q-Actin standardized cucumber extract using general toxicity studies in rats and a battery of genotoxicity assays. The general toxicity was evaluated by conducting acute and repeated dose toxicity studies (up to 90 days) in Sprague Dawley rats. Genotoxicity of Q-Actin was evaluated by Ames test*, in vitro* chromosomal aberration assay in CHO-K1 cell line, and *in vivo* micronucleus test in mice. All studies were conducted at Vipragen Biosciences Pvt. Ltd., Mysore, Karnataka, India.

## 2 Materials and methods

### 2.1 Test material

Q-Actin is a 12.5:1 aqueous extract of *Cucumis sativus* (cucumber) fruit. The extract, is manufactured under current good manufacturing practices (cGMPs) using a patented process that concentrates the *ido*-BR1 iminosugar. The test material used in the assay met the specifications as shown in Table 1.

**Table 1:** Specifications of standardized cucumber extract Q-Actin.

Parameter	Specification	Assay Method
Description	Dark brown, non-fibrous powder	Organoleptic
Odor	Characteristic herbal	Organoleptic
Taste	Slightly bitter	Organoleptic
Loss on drying	<6%	USP <731>
Solubility (1 g/300 ml water)	80%	USP General Chapter
pH (1 g/100 ml water)	4.0 – 6.5	pH meter
Particle size	98% thru 80 mesh	USP <786>
Tap density	0.4 – 0.95 g/cc	USP <616>
Bulk density	0.3 – 0.8 g/cc	USP <616>
*Chemical assay*		
Iminosugar Ido-BR-1	>1% but no more than 2% on dried basis	GCMS

*Heavy metals*		
Lead	<0.5 ppm	ICP-MS
Mercury	<0.2 ppm	ICP-MS
Cadmium	<0.5 ppm	ICP-MS
Arsenic	<0.5 ppm	ICP-MS

*Microbiological assays*		
Total plate count (CFU/g)	Less than 1000	USP <2021>
Yeast and mold (CFU/g)	Less than 100	USP <2021>
*E. coli* (CFU/g)	Negative	USP <2022>
*Salmonella* (CFU/375 g)	Negative	USP <2022>
*S. aureus* (CFU/25 g)	Negative	USP <2022>
Total Coliforms (CFU/g)	Less than 3	FDA-BAM

*Pesticide assays*		USP <561>
Organochlorine	Negative	
Organophosphorus	Negative	
Organonitrogen	Negative	
*N*-Methyl carbamates	Negative	
Aflatoxins	Negative	

FDA-BAM = US Food and Drug Administration Bacteriological Analytical Manual; GCMS = gas chromatography-mass spectrometry; ICP-MS = inductively coupled plasma mass spectrometry; USP = United States Pharmacopeia.

#### 2.1.1 Test item preparation for oral (gavage) toxicity studies

Q-Actin, as defined in 2.1 and meeting the specifications identified in Table 1, was used in the study. Milli Q water served as the vehicle for formula preparation. The formulation of the test item was prepared shortly before dose administration and homogeneity was maintained during the administration using a magnetic stirrer.

### 2.2 Acute oral (gavage) toxicity study

The acute oral (gavage) toxicity study was conducted in compliance with OECD test guideline 423 [13]. In two separate experiments, 3 female Sprague-Dawley rats (9–10 weeks of age; body weights 126–161 g) received a single dose of Q-Actin at 2000 mg/kg, dissolved in Milli Q water. Following the dose administration, animals were observed for mortality, clinical signs of toxicity, changes in body weight for 14 days and gross pathology at the end of experiment.

### 2.3 Repeat-dose oral (gavage) toxicity studies (28 and 90 day with recovery)

#### 2.3.1 Study design

The 28 and 90 day oral (gavage) repeat dose toxicity studies were performed in compliance with OECD test Guideline 407 [14] and 408 [15], respectively. The studies were approved by the Institutional Animals Ethics Committee of the test facility. The test facility is approved by the Committee for the Purpose of Control and Supervision on Experiments on Animals (CPCSEA), INDIA. The studies were performed following all ethical practices as laid down in the guidelines for animal care.

#### 2.3.2 Animals

Male and female Sprague-Dawley rats (Source: Adita Biosys Private Limited, Tumkuru, India) were acclimatized to laboratory conditions for 5 days after veterinary examination.

In the 28-day study, a total of 72 animals (36/sex) were stratified based on body weight and randomly assigned into 6 groups (6/sex/group). The body weight at the beginning of the treatment were 74.61–115.32 g for males and 69.15–101.2 g for females.

In the 90-day study, a total of 100 animals (50/sex) were stratified based on body weight and randomly assigned into 6 groups. Ten rats/sex/group were assigned to main groups and five rats/sex/group were assigned to recovery groups. The body weight at the beginning of the treatment was 84.38–112.50 g for males and 86.11–108.46 g for females.

All animals were housed under standard laboratory conditions and in accordance with CPCSEA guidelines [16]. The animals were fed *ad libitum* with Teklad Feed manufactured by ENVIGO USA throughout the experimental periods. Reverse osmosis water was provided *ad libitum* throughout the experimental periods.

#### 2.3.3 Treatment

The selected dose levels of Q-Actin for both the 28-day and 90-day repeated dose study were 0, 250, 500 and 1000 mg/kg/day. The dose levels were selected based on the results of acute oral (gavage) toxicity, where the dose level of 2000 mg/kg was well tolerated without any toxicity. The total dose volume of 10 ml/kg was used in both studies.

In the 28-day study, the highest dose, 1000 mg/kg was dosed to 5 rats/sex for one week in a pretest of this study to confirm the tolerability of the dose. There were no adverse effects observed in this pretest (data not shown). The rats in the main study were treated orally (gavage) once daily with Q-Actin cucumber extract for 28 consecutive days at dose levels of 0 for G1 and G1R groups (vehicle control and vehicle control recovery), 250 mg/kg bw (G2 low dose group), 500 mg/kg bw (G3 mid dose group) and 1000 mg/kg bw for the G4 and G4R (high dose and high dose recovery) groups. Recovery groups were maintained for an additional 14 days following the end of the treatment period.

In the 90-day study, the rats in the main study were treated orally (gavage) once daily with Q-Actin for 90 consecutive days at dose levels of 0 for G1 and G1R groups (vehicle control and vehicle control recovery), 250 mg/kg bw (G2 low dose group), 500 mg/kg bw (G3 mid dose group) and 1000 mg/kg bw for the G4 and G4R (high dose and high dose recovery) groups. Recovery groups were maintained for an additional 28 days following the end of the treatment period.

#### 2.3.4 Parameters

##### 2.3.4.1 Mortality, clinical examination, body weight and feed consumption

All animals were observed for morbidity, mortality and clinical signs of toxicity during the study periods. On the first three days of dosing, post dose observations were made twice daily at hourly intervals. Subsequent post dose observations were recorded once approximately one hour after dose administration. Detailed clinical signs observation (including but not limited to changes in skin, fur, eyes, mucous membranes, occurrence of secretions and autonomic activity and changes in gait, posture and response to handling, presence of clonic or tonic movements or bizarre behavior) was made once a week during the study period before dosing on the observation day. Body weights of all animals were recorded once a week both in main and recovery periods of studies. Feed consumption was measured once weekly during dosing and recovery period except during the terminal sacrifice of main and recovery animals.

##### 2.3.4.2 Functional observation battery and ophthalmoscopic examination

Functional observation battery was not recorded for the animals in either study, since there were no test item related clinical signs observed in the detailed clinical signs observation. Ophthalmological examination of each animal was conducted by a trained veterinarian prior to the start of treatment and at the end of the dosing period. Prior to examination, mydriasis was induced using a 1% solution of Tropicamide.

##### 2.3.4.3 Clinical pathology- hematology, clinical chemistry and urinalysis

In the sub-acute and sub-chronic toxicity studies, blood samples for hematology, clinical biochemistry, and urine samples were collected from both main and recovery animals at the end of the experiment. Individual animals were kept in the metabolic cages for urine collection at the end of the main and recovery period of the studies. Animals were fasted overnight but allowed water *ad libitum* prior to blood sampling. Blood samples were collected from the retro-orbital plexus using a micro-hematocrit heparinized glass capillary tube under isoflurane/oxygen anesthesia for hematological and clinical chemistry evaluation. For blood sampling, a tube containing dipotassium ethylenediamine tetra acetic acid (K2-EDTA 10%) for hematology and the other tube without the anti-coagulant for clinical chemistry analysis, respectively were used.

The following hematological parameters were evaluated: total leukocyte count (WBC), erythrocyte count (RBC), hemoglobin (HGB), hematocrit (HCT), platelet count (PLT), mean corpuscular volume (MCV), mean corpuscular hemoglobin (MCH), mean corpuscular hemoglobin concentration (MCHC), differential leucocytes count (DLC). Reticulocyte count were also measured in the 28-day study. Blood smears were prepared using standard techniques and stained with Giemsa for enumerating the differential leucocytes cells. A total of 100 cells were counted per animal and total number of lymphocytes (LYM), monocytes (MON), neutrophils (NEU), eosinophils (EOS) and basophiles (BAS) were counted. The following clinical chemistry parameters were studied, total protein (TB), albumin (ALB), alanine aminotransferase (ALT), aspartate aminotransferase (AST), alkaline phosphatase (ALP), glucose, total cholesterol (TCHO), creatinine (CRE), serum urea (URE), sodium, potassium and calcium. Urine analysis included appearance, volume, specific gravity, pH, leucocytes, protein, glucose, and erythrocytes were estimated in both the 28- and 90-day studies. Additional parameters of nitrate (NIT), ketones (KET), urobilinogen (UBG) and bilirubin (BIL) were estimated in the 28-day study.

##### 2.3.4.4 Pathology- gross necropsy, organ weights, tissue collection and histopathology

At the end of Q-Actin treatment and recovery periods, all animals in both studies were subjected to complete necropsy which included careful examination of the external surface of the body, all orifices, the cranial, thoracic, and abdominal cavities and their contents. Animals were fasted overnight with access to water and anesthetized with carbon dioxide asphyxiation and exsanguinated. The individual organs, liver, kidney, spleen, adrenal, thymus, heart, brain, prostate glands, testes, epidydimis, ovaries, uterus were collected, trimmed of any adherent tissue and weighed as soon as the dissection was complete. A total of more than thirty organs and tissues were collected and fixed in 10% neutral buffered formalin for microscopic examination. For histopathology, examinations were carried out on the preserved organs of the vehicle control (G1) and high dose (G4) rats in both studies. Tissues were processed, embedded in paraffin, sectioned at 4–5 micrometers and stained with hematoxylin and eosin. The unused tissues were archived.

#### 2.3.5 Statistical analysis

Statistical analysis in both the 28- and 90-day studies was performed using the StatPlus analysis program. All data was checked for normality with Shapiro-Wilk normality test, and data for each group of animals was subjected to analysis of variance (ANOVA). Values were given as mean ± standard deviation (SD). Dunnet’s *t*-test was used to compare the differences between treated and control groups and statistical significances of differences were calculated with one-way ANOVA. All analysis and comparisons were evaluated at 95% level of confidence (p ≤ 0.05).

### 2.4 Mutagenicity studies

#### 2.4.1 Bacterial reverse mutation test (Ames assay)

The mutagenicity potential of Q-Actin was evaluated in the Bacterial Reverse Mutation Assay in accordance with OECD test guideline 471 [17] and ICH guidelines. *Salmonella typhimurium* strains TA98, TA100, TA102, TA1535 and TA1537 (Moltox, USA) were used in this study. The study was conducted in the presence and absence of external metabolic activation system, S9 rat liver metabolic activation (Aroclor 1254-induced; Moltox, USA). The mutagenic potential was evaluated using two different procedures, namely preincubation and plate incorporation methods. Milli Q water was used as a vehicle and Q-Actin completely dissolved in vehicle and formed a uniform suspension at 50 mg/ml. For each concentration of test item, vehicle and positive control, triplicate plates were used in the study. Positive controls were used for both the presence and absence of metabolic activation system. The selected positive controls were 2-nitroflurene, sodium azide, cumene hydroperoxide and acridine mutagen ICR 191 for TA98, TA1535 & TA100, TA102 and TA1537, respectively. In case of study with metabolic activation, 2-aminoantracene was used as a positive control.

A preliminary study to evaluate the cytotoxicity of Q-Actin and also to select the concentrations to be used in the main study was conducted using the preincubation method with *Salmonella typhimurium* strain TA100. The tester strains were revived by adding 10 μl to Erlenmeyer flasks containing 20 ml of Oxoid nutrient broth #2 and were incubated for 14 h at 37 ± 2 °C in a shaking incubator at 110 ± 10 rpm. The approximately total viable cell count used for each experiment was 10^9^ cells/ml. A total of seven concentrations at two-fold intervals were selected and the selected concentrations were 39, 78, 156, 625, 1250, 2500 and 5000 μg/plate. The cytotoxicity was evaluated by observing the reduction in the number of spontaneous revertant or a clearing or diminution of the bacterial background lawn.

There was no precipitation of Q-Actin observed in any of the tested concentrations and no significant decrease in the number of revertant colonies were observed in comparison to the concurrent vehicle control. Based on these observations, concentrations of 50, 150, 500, 1500 and 5000 μg/plate were selected for the main study.

The main assay was conducted using the pre-incubation method and plate incorporation method using *Salmonella typhimurium* strains TA98, TA100, TA102, TA1535, and TA1537 in the presence and absence of S9 rat liver metabolic activation. The detailed procedures for preincubation and plate incorporation are described in the OCED guideline, OECD 471.

For both methods in the main study, plating was achieved by adding 0.1 ml of the *S. typhimurium* tester strains, 0.1 ml of Q-Actin solution or vehicle or positive control, and 0.5 ml of S9-mix or phosphate buffer solution. For the pre-incubation method, the test item solution was preincubated with the test strain and sterile buffer or S-9 mix for 20 min at 37 °C prior to mixing with 2 ml of overlay agar and pouring onto the surface of a minimal glucose agar plate. For the plate incorporation method, the test item solution was mixed with the test strain and sterile buffer or S-9 mix and 2 ml of overlay agar and poured onto the surface of a minimal glucose agar plate.

Each strain/dose level/vehicle/positive control in the plate incorporation method were performed in triplicate. Following solidification, the plates were incubated at 37 ± 2 °C for 48 h. The plates were examined manually for cytotoxicity by means of reduction in the background lawn and number of revertant colonies. A mutagenic effect was concluded if the number of revertant colonies in the test dose was more than twice (TA98, TA100 and TA 102) or three times (TA1535 and TA1537) the count of the corresponding control. The study was acceptable if the positive control substances produced a significant increase in mutant colony frequencies, the spontaneous reversion rates in the negative control fall within three times the standard deviation of the means of normal rate and no more than 5% of plates are lost through contamination.

#### 2.4.2 *In vitro* chromosomal aberration assay

The potential of Q-Actin cucumber extract to induce chromosomal aberrations was evaluated in Chinese hamster ovary cells (CHO-K1). The assay was performed in compliance with OECD guideline 473 [18]. CHO-K1 cells were procured from NCCS, Pune, India and cultured in Ham’s F12 nutrient media supplemented with fetal bovine serum (FBS).

Cytotoxicity of Q-Actin cucumber extract was determined using five doses (0.21, 0.42, 0.83, 1.67 and 3.33 mg/ml) in CHO-K1 cells by evaluating the changes in the morphology of cells. The culture media served as the negative control. Cultured cells were trypsinized and approximately 1 × 10^5^ cells/well in duplicate were seeded into 12 well plates and incubated for 24 h at 37 °C with 5 ± 1% CO_2_ and 95% humidity. Three sets of plates per concentration were made, namely first set was prepared with metabolic activation system consisting of 0.1 ml of S-9 mix to achieve a concentration of 1% (v/v) in each well; second and third set, without metabolic activation (Sets 2 and 3) included 0.1 ml of F12 with 5% FBS. Treatments in Sets 1 and 2 were terminated after 3 h and by removing the test solution, washing with phosphate buffered saline (PBS) twice and re-fed with 3 ml of F12 10% FBS and incubated for 17 h. Medium was aspirated from all three sets around 20 h and 20 min after the start of treatment, cells were trypsinized and suspended with 1 ml of F12 5% FBS. Cell suspensions were pooled from the duplicate cells and cell count were determined by using a Hemocytometer. The effect of Q-Actin on cell multiplication was determined using the Relative Increase in Cell Count (RICC) using the following formula:

$$\text{RICC}\left(\%\right)=\frac{(\text{Increase in number of cells in treated cultures}\left(\text{final}–\text{ starting}\right)}{(\text{Increase in number of cells in control cultures}\left(\text{final -starting}\right)\text{.}}$$

The non-cytotoxic doses of 0.83, 1.67, and 3.33 of Q-Actin cucumber extract were selected for the chromosome aberration test, with or without S9 in short term (3 h) and without S9 in long-term (20 h) exposure. Cell seeding, incubation and treatment procedure was identical to that used in the cytotoxicity test. Positive controls included 0.0003 mg/ml Mitomycin C (-S9) and 0.006 mg/ml cyclophosphamide monohydrate (+S9). Before harvesting the cells, 0.2 μg/ml of Colchicine was added to each concentration, incubated for two hours then trypsinized, incubated in KCl for 15 min and fixed with acetic acid and methanol. Slides of the cells were prepared and stained using 5% Giemsa for microscopic evaluation. Three hundred well-spread metaphases were scored per test concentration, positive and negative control. The cells scored contained a number of centromeres equal to the modal number ± 2. Chromatid- and chromosome-type aberrations were recorded separately and classified by sub-types (breaks, exchanges). Concurrent measures of cytotoxicity for all treated, negative and positive control cultures in the main assay were recorded.

#### 2.4.3 *In vivo* micronucleus test

The potential of orally administered Q-Actin cucumber extract to induce micronuclei in polychromatic erythrocytes (PCE) in the bone marrow of male and female Swiss albino mice was evaluated in accordance with OECD Guideline 474 [19]. The preliminary range finding toxicity study utilized two groups (5/sex) of mice orally administered with a single 2000 mg/kg bw dose of Q-Actin in Milli Q water by oral (gavage) route. Animals were observed for clinical signs of toxicity at approximately 0.5, 1, 2, 4, 24 and 48 h post administration. There were no signs of toxicity observed at 2000 mg/kg and this dose was used as a highest dose for the main study.

In the main study, a total of seventy animals aged 8-10-week-old (22–28 g) were equally divided into 7 groups consisting of 5 animals/sex. The body weight of the animals was measured on day 0 and after 24 and 48 h of treatment. The animals received a single oral dose (volume 10 ml/kg bw) of vehicle (G1), 250 (G2), 500 (G3), and 1000 (G4), 1000 (G5) mg/kg bw of Q-Actin. Animals from the positive control groups G6 and G7 received single doses of cyclophosphamide monohydrate (30 mg/kg bw) and methyl methane sulfonate (50 mg/kg bw) intraperitoneally, respectively.

Animals in all dose groups were examined for signs of toxicity at approximately 1, 2, 4 and 24 h and up to 48 h for the high dose group G5. All animals were subjected to gross pathological evaluation following terminal sacrifice. The animals from control, low and mid dose groups were euthanized using CO_2_ after 24 h of treatment and high dose animals were sacrificed after 48 h. The femora were removed, the epiphyses were cut off and the marrow flushed out with FBS. The collected bone marrow was centrifuged, the supernatant discarded and a small drop of the re-suspended cell pellet was spread on a slide, air dried and stained with Giemsa. A total of two slides were prepared from each bone marrow sample and at least 4000 PCE were analyzed per animal for micronuclei and the ratio of polychromatic erythrocytes to the total number of erythrocytes were recorded for each animal. The data was subjected to statistical analysis (ANOVA and Dunnett’s *t*-test and non-parametric Mann-Whitney test).

## 3 Results

### 3.1 Acute oral (gavage) toxicity study

Single oral (gavage) administration of Q-Actin at 2000 mg/kg did not cause mortality or clinical signs of toxicity in female SD rats. There were no Q-Actin related gross pathological changes observed at the end of the 14-day observation period. Based on the results, the LD_50_ of Q-Actin is greater than 2000 mg/kg body weight and is unlikely to be toxic and can be classified under Category 5 per the Globally Harmonized Classification System [20].

### 3.2 28-Day repeat-dose oral (gavage) toxicity study

The treatment with Q-Actin at dose levels of 250, 500 and 1000 mg/kg body weight per day to Sprague Dawley rats for 28 consecutive days had no noteworthy effects on general health of the animals, body weight, net body weight gains, feed consumption, hematology, clinical chemistry, serum electrolytes, urine parameters, fasting body weights, organ weights, macro and microscopic lesions in both sexes at all treated groups (data not shown). Considering the results observed, the evaluated No Observed Adverse Effect Level (NOAEL) is determined to be 1000 mg/kg body weight per day for Q-Actin under the test conditions and doses employed.

### 3.3 90-Day repeat-dose oral (gavage) toxicity study

#### 3.3.1 Clinical examination, body weight and feed consumption

There was no treatment related clinical signs or mortality observed during the course of the study in the animals of any group. Statistically significant difference in body weight and body weight gain were observed as follows: increase of net body weight gain in G3 males and decrease in G4R males during Day 64–71; decrease in body weight gain in G2 and G4 females during day 01–08; increase in net body weight gain in G4R females (compared to G1R females) in days 29–36 and 43–50; and decrease in net body weight gain in G4R females in days 01–08 and 92–99 (data not shown). None of the differences in body weight or body weight gain were considered to be toxicologically important because there was no dose-response effect and there were no significant differences at the end of the study period. Therefore, these changes were not considered as treatment related. A statistically significant increase in feed consumption (day 15–22) was observed in the G4R males and was considered as incidental in nature.

#### 3.3.2 Ophthalmoscopic examination

Ophthalmological examination did not reveal any abnormalities at the end of the treatment period (data not shown).

#### 3.3.3 Clinical pathology- hematology, clinical chemistry and urinalysis

There was no Q-Actin treatment related change in the hematological parameters in male and female rats, except an isolated incidence of statistically significant increase in lymphocytes (LYM) in G2 females (Table 2, Table 3). As the increase in LYM was associated with only one sex, and did not show a dose-response effect, the finding was considered incidental and not related to the test article.

**Table 2:** Effect of 90-day oral administration of a standardized cucumber extract (Q-Actin) on hematological parameters in male rats (n = 50).

Parameter	Units	Group and Dose (mg/kg/day)					
		G1 (0 mg) (n = 10)	G2 (250 mg) (n = 10)	G3 (500 mg) (n = 10)	G4 (1000 mg) (n = 10)	G1R (0 mg) (n = 5)	G4R (1000 mg) (n = 5)
WBC	10^3^ cell/mm^3^	9.53 ± 2.74	8.79 ± 3.01	9.69 ± 2.21	10.46 ± 3.81	8.88 ± 1.95	8.74 ± 1.89
RBC	10^6^ cell/mm^3^	8.46 ± 0.59	8.49 ± 0.38	8.32 ± 0.34	8.50 ± 0.55	8.46 ± 0.35	8.84 ± 0.40
HGB	g/dL	14.48 ± 0.85	14.74 ± 0.72	14.59 ± 0.65	14.32 ± 0.51	14.62 ± 0.26	14.60 ± 0.87
HCT	%	43.18 ± 2.49	43.79 ± 1.94	43.19 ± 1.85	42.74 ± 1.97	44.30 ± 0.73	44.54 ± 2.39
PLT	10^3^ cell/mm^3^	604.3 ± 80.40	613.89 ± 80.6	655.1 ± 88.98	579.4 ± 85.39	616.4 ± 124.03	569.00 ± 64.00
MCV	μm^3^	51.00 ± 2.75	51.67 ± 1.66	51.80 ± 1.32	50.30 ± 1.89	52.60 ± 2.07	50.60 ± 1.52
MCH	pg	17.14 ± 0.72	17.37 ± 0.69	17.55 ± 0.65	16.88 ± 0.84	17.32 ± 0.70	16.52 ± 0.72
MCHC	g/dL	33.52 ± 0.62	33.66 ± 0.62	33.83 ± 0.73	33.51 ± 0.74	33.02 ± 0.45	32.72 ± 0.47
LYM	%	68.70 ± 3.68	61.22 ± 12.41	67.00 ± 2.94	65.60 ± 4.20	68.40 ± 2.70	72.78 ± 5.34
MON	%	2.90 ± 0.74	2.89 ± 0.60	2.80 ± 0.92	3.00 ± 0.82	3.00 ± 0.71	2.88 ± 0.75
NEU	%	26.10 ± 3.63	33.44 ± 11.08	29.30 ± 4.45	29.40 ± 3.81	26.60 ± 2.41	22.94 ± 3.61
EOS	%	2.30 ± 0.82	2.44 ± 1.13	1.90 ± 0.57	2.00 ± 0.00	2.00 ± 0.00	2.20 ± 0.45
BAS	%	0.0 ± 0.0	0.0 ± 0.0	0.0 ± 0.0	0.0 ± 0.0	0.0 ± 0.0	0.0 ± 0.0

Values are mean ± SD for 10 rats in each group per sex G1, G2, G3 & G4 & 5 rats in G1R and G4R group per sex; BAS = basophiles; EOS = eosinophils; HCT = hematocrit; HGB = hemoglobin; LYM = lymphocytes; MCH = mean corpuscular hemoglobin; MCHC = mean corpuscular hemoglobin concentration; MCV = mean corpuscular volume; MON = monocytes; NEU = neutrophils; PLT = platelet count; RBC = erythrocyte count; WBC = total leukocyte count; No statistically significant differences between groups.

**Table 3:** Effect of 90-day oral administration of a standardized cucumber extract (Q-Actin) on hematological parameters in female rats (n = 50).

Parameter	Units	Group and Dose (mg/kg/day)					
		G1 (0 mg) (n = 10)	G2 (250 mg) (n = 10)	G3 (500 mg) (n = 10)	G4 (1000 mg) (n = 10)	G1R (0 mg) (n = 5)	G4R (1000 mg) (n = 5)
WBC	10^3^ cell/mm^3^	8.71 ± 2.04	8.29 ± 1.50	9.97 ± 2.52	8.32 ± 2.66	5.78 ± 1.61	7.25 ± 3.77
RBC	10^6^ cell/mm^3^	7.74 ± 0.25	7.78 ± 0.25	7.79 ± 0.32	7.76 ± 0.41	8.23 ± 3.19	7.16 ± 1.96
HGB	g/dL	15.25 ± 0.67	15.06 ± 0.51	15.10 ± 0.44	15.45 ± 0.41	18.46 ± 10.87	12.25 ± 3.22
HCT	%	41.58 ± 1.30	41.34 ± 1.25	41.16 ± 1.16	41.73 ± 1.37	43.28 ± 17.16	36.66 ± 9.70
PLT	10^3^ cell/mm^3^	558.40 ± 79.89	594.1 ± 57.64	574.9 ± 79.83	597.10 ± 76.91	585.60 ± 228.51	574.09 ± 172.65
MCV	μm^3^	53.90 ± 2.08	53.30 ± 1.83	52.80 ± 1.32	53.70 ± 2.11	51.80 ± 20.47	45.91 ± 12.50
MCH	pg	19.68 ± 0.91	19.36 ± 0.78	19.42 ± 0.89	19.94 ± 0.79	17.34 ± 6.81	15.37 ± 4.21
MCHC	g/dL	36.61 ± 0.83	36.43 ± 0.49	36.73 ± 1.01	37.03 ± 0.53	33.42 ± 13.43	30.10 ± 8.17
LYM	%	66.50 ± 3.81	70.80* ± 3.08	67.70 ± 2.98	69.20 ± 3.65	66.60 ± 25.78	57.96 ± 15.92
MON	%	3.30 ± 0.67	2.90 ± 0.88	3.10 ± 0.88	2.80 ± 0.92	3.00 ± 1.06	3.01 ± 1.25
NEU	%	28.40 ± 4.33	24.50 ± 2.72	27.40 ± 2.50	26.60 ± 3.60	28.40 ± 10.31	26.55 ± 8.11
EOS	%	1.80 ± 0.42	1.80 ± 0.63	1.80 ± 0. 42	1.40 ± 0.52	2.00 ± 0.88	2.15 ± 0.79
BAS	%	0.00 ± 0.00	0.00 ± 0.00	0.00 ± 0.00	0.00 ± 0.00	0.00 ± 0.00	0.00 ± 0.00

Values are mean ± SD for 10 rats in each group per sex G1, G2, G3 & G4 & 5 rats in G1R and G4R group per sex; BAS = basophiles; EOS = eosinophils; HCT = hematocrit; HGB = hemoglobin; LYN = lymphocytes; MCH = mean corpuscular hemoglobin; MCHC = mean corpuscular hemoglobin concentration; MCV = mean corpuscular volume; MON = monocytes; NEU = neutrophils; PLT = platelet count; RBC = erythrocyte count WBC = total leukocyte count; *= p ≤ 0.05.

Clinical chemistry results showed a statistically significant decrease of serum sodium in G3 female and increase in G4 females. These changes were not toxicologically relevant as the changes were of low magnitude and there were no changes in the recovery group compared to control (Table 4). Treatment of Q-Actin caused a statistically significant increase of potassium in G3 and G4 females compared to the control at the end of the 90-day treatment period (Table 4). Even though there is a dose dependent increase in the potassium level, the observed increase is not considered biologically significant as the values are within the testing laboratory reference range of 4.1–6.1 mmol/l and the changes are not observed at the end of the recovery period. Furthermore, there were no changes in other electrolyte values. Based on these observations, the increase in serum potassium level in females is not considered as an adverse effect. There were no statistically significant changes in any urinalysis parameters measured in any of the groups (data not shown).

**Table 4:** Effect of 90-day oral administration of a standardized cucumber extract (Q-Actin) on clinical chemistry parameters in male (n = 50) and female (n = 50) rats.

Parameter	Units	Group and Dose (mg/kg/day)					
		G1 (0 mg)	G2 (250 mg)	G3 (500 mg)	G4 (1000 mg)	G1R (0 mg)	G4R (1000 mg)
		(n = 10)	(n = 10)	(n = 10)	(n = 10)	(n = 5)	(n = 5)
**Males**							
TP	g/L	77.67 ± 2.24	79.52 ± 2.49	78.96 ± 4.48	78.81 ± 1.74	71.70 ± 14.54	83.92 ± 7.04
ALB	g/L	38.58 ± 1.52	39.79 ± 2.37	38.50 ± 1.55	37.96 ± 1.86	40.27 ± 4.56	39.52 ± 5.70
ALT	U/L	67.29 ± 18.02	63.48 ± 11.75	65.35 ± 17.05	88.79 ± 64.57	66.36 ± 10.66	71.28 ± 15.26
AST	U/L	121.56 ± 18.54	137.37 ± 8.03	136.43 ± 18.86	128.35 ± 17.28	117.74 ± 5.50	124.28 ± 14.29
ALP	U/L	141.63 ± 36.57	132.03 ± 64.89	113.45 ± 52.72	125.36 ± 31.40	123.50 ± 34.42	127.80 ± 48.92
Glucose	mg/dL	111.04 ± 21.38	95.42 ± 20.53	100.38 ± 27.39	102.06 ± 17.81	129.34 ± 34.51	133.64 ± 15.81
TCHO	mg/dL	70.20 ± 10.19	66.23 ± 8.59	67.67 ± 9.52	67.80 ± 10.86	66.95 ± 22.46	74.47 ± 15.98
CRE	mg/dL	0.35 ± 0.07	0.35 ± 0.07	0.37 ± 0.05	0.40 ± 0.13	0.35 ± 0.18	0.46 ± 0.10
URE	mg/dL	37.46 ± 5.53	36.91 ± 4.03	38.36 ± 6.36	38.62 ± 5.56	38.77 ± 14.34	42.42 ± 3.96
TB	mg/dL	0.14 ± 0.06	0.09 ± 0.06	0.10 ± 0.14	0.09 ± 0.04	0.05 ± 0.07	0.05 ± 0.02
Na	mmol/L	133.40 ± 3.41	133.89 ± 3.33	133.30 ± 1.64	133.10 ± 2.28	152.80 ± 13.46	152.80 ± 10.52
K	mmol/L	5.82 ± 1.07	6.56 ± 1.02	5.99 ± 0.69	6.08 ± 1.15	6.30 ± 1.42	5.94 ± 0.34
Ca	mmol/L	1.18 ± 0.08	1.20 ± 0.05	1.19 ± 0.03	1.17 ± 0.05	0.93 ± 0.21	1.00 ± 0.09

**Females**							
TP	g/L	83.46 ± 3.39	81.57 ± 2.95	82.46 ± 5.22	82.10 ± 3.13	76.14 ± 12.45	83.26 ± 4.38
ALB	g/L	43.16 ± 3.15	45.12 ± 2.28	43.41 ± 2.98	42.79 ± 8.71	47.64 ± 4.15	45.91 ± 3.96
ALT	U/L	78.13 ± 49.19	57.90 ± 14.46	61.92 ± 17.72	60.18 ± 18.05	54.94 ± 7.24	58.66 ± 12.50
AST	U/L	139.16 ± 60.30	117.00 ± 17.96	129.08 ± 20.65	130.22 ± 35.27	105.18 ± 16.58	121.12 ± 21.14
ALP	U/L	125.46 ± 38.63	112.59 ± 37.26	115.25 ± 48.10	111.42 ± 49.41	88.72 ± 36.58	73.00 ± 31.00
Glucose	mg/dL	112.29 ± 12.46	97.36 ± 13.09	103.20 ± 16.38	101.58 ± 21.38	122.31 ± 15.83	132.43 ± 20.47
TCHO	mg/dL	77.72 ± 8.71	80.03 ± 11.29	72.43 ± 14.06	79.29 ± 14.52	71.22 ± 19.28	68.71 ± 14.39
CRE	mg/dL	0.41 ± 0.10	0.46 ± 0.11	0.48 ± 0.05	0.51 ± 0.07	0.37 ± 0.19	0.48 ± 0.03
URE	mg/dL	42.36 ± 6.37	41.18 ± 5.60	38.23 ± 8.28	40.24 ± 5.09	39.83 ± 22.28	45.32 ± 4.08
TB	mg/dL	0.11 ± 0.11	0.08 ± 0.07	0.08 ± 0.07	0.07 ± 0.06	0.06 ± 0.06	0.04 ± 0.05
Na	mmol/L	140.20 ± 2.30	139.90 ± 1.85	138.50* ± 1.51	141.60* ± 1.51	155.60 ± 8.11	160.40 ± 6.35
K	mmol/L	4.94 ± 0.25	5.24 ± 1.02	5.59* ± 0.55	5.84* ± 0.56	5.28 ± 0.71	5.36 ± 0.90
Ca	mmol/L	1.24 ± 0.03	1.26 ± 0.04	1.26 ± 0.04	1.25 ± 0.07	1.22 ± 0.13	1.20 ± 0.08

Values are mean ± SD for 10 rats in each group per sex G1, G2, G3 & G4 & 5 rats in G1R and G4R group per sex; ALB = albumin; ALP = alkaline phosphatase; ALT = alanine aminotransferase; AST = alanine aminotransferase; CRE = creatinine; URE = serum urea; TP = total protein; TCHO = total cholesterol; TB = total bilirubin *= p ≤ 0.05.

#### 3.3.4 Pathology- gross necropsy, organ weights, and histopathology

There were no treatment-related changes in absolute and relative organ weights in male and female rats. In the G4R females, an isolated incidence of statistically significant increase in absolute organ weights of the kidney, spleen and thymus (Table 5) was observed and a statistically significant decrease in relative organ weight of the heart (data not shown) were observed. All other values on absolute organ weights were comparable among the groups (Table 5). The statistical analysis was carried out for the two different groups, with the main treatment group (G2, G3 and G4) compared to the G1 control group and the G4R group compared to the G1R control group. Histopathology revealed no abnormality of the adrenal glands, thymus or spleen. Histopathology of the kidneys showed moderate cellular infiltration in two areas in the pelvis region in one animal in the G1 group and two animals in the G4 group showed mild dilation of tubular epithelium and glomerular distension. No other animals of either male and female in the G1 and G4 groups showed any abnormality. Histopathological examination showed no treatment related changes in any of the organs examined. As there were no correlated findings in the histopathology of the kidney, spleen, thymus and adrenal glands, the differences in absolute organ weights and relative organ weights are considered unrelated to treatment.

**Table 5:** Effect of 90-day oral administration of a standardized cucumber extract (Q-Actin) on absolute organ weights (g) in male (n = 50) and female (n = 50) rats.

**Organs**	**Group**					
	G1 (0 mg)	G2 (250 mg)	G3 (500 mg)	G4 (1000 mg)	G1R (0 mg)	G4R (1000 mg)
	(n = 10)	(n = 10)	(n = 10)	(n = 10)	(n = 5)	(n = 5)
**Males**						
Liver	8.89 ± 1.47	8.99 ± 1.30	8.82 ± 1.14	8.58 ± 1.99	9.18 ± 1.96	8.99 ± 1.85
Kidneys	1.82 ± 0.27	1.84 ± 0.27	1.93 ± 0.24	1.78 ± 0.18	1.99 ± 0.36	2.04 ± 0.31
Adrenals	0.05 ± 0.01	0.06 ± 0.02	0.06 ± 0.02	0.06 ± 0.01	0.05 ± 0.02	0.05 ± 0.01
Spleen	1.10 ± 0.25	1.06 ± 0.13	1.07 ± 0.14	1.07 ± 0.17	1.07 ± 0.09	1.03 ± 0.25
Heart	0.99 ± 0.11	1.07 ± 0.12	1.08 ± 0.14	1.02 ± 0.15	1.12 ± 0.19	0.98 ± 0.15
Thymus	0.37 ± 0.16	0.30 ± 0.06	0.35 ± 0.14	0.33 ± 0.10	0.30 ± 0.15	0.34 ± 0.06
Brain	1.82 ± 0.17	1.84 ± 0.14	1.89 ± 0.13	1.84 ± 0.08	1.90 ± 0.12	1.90 ± 0.26
Testes	2.97 ± 0.24	3.02 ± 0.22	3.09 ± 0.20	3.07 ± 0.22	3.07 ± 0.29	3.10 ± 0.25
Epididymides	1.22 ± 0.11	1.35 ± 0.26	1.32 ± 0.15	1.43 ± 0.30	1.38 ± 0.32	1.31 ± 0.19

**Females**						
Liver	6.46 ± 0.85	5.72 ± 0.61	6.38 ± 0.61	5.93 ± 0.87	5.63 ± 0.82	6.39 ± 0.61
Kidneys	1.29 ± 0.17	1.12 ± 0.15	1.21 ± 0.10	1.19 ± 0.20	1.20 ± 0.13	1.35*± 0.06
Adrenals	0.07 ± 0.01	0.06 ± 0.02	0.06 ± 0.02	0.06 ± 0.02	0.07 ± 0.02	0.06 ± 0.02
Spleen	0.83 ± 0.14	0.78 ± 0.14	0.94 ± 0.15	0.80 ± 0.11	0.74 ± 0.11	0.89* ± 0.09
Heart	0.88 ± 0.10	0.82 ± 0.15	0.84 ± 0.15	0.85 ± 0.16	0.77 ± 0.08	0.75 ± 0.05
Thymus	0.29 ± 0.08	0.28 ± 0.10	0.26 ± 0.08	0.25 ± 0.08	0.25 ± 0.05	0.33*± 0.05
Brain	1.62 ± 0.23	1.75 ± 0.06	1.74 ± 0.12	1.81 ± 0.07	1.65 ± 0.32	1.85 ± 0.07
Ovaries	0.14 ± 0.04	0.15 ± 0.05	0.13 ± 0.04	0.13 ± 0.05	0.13 ± 0.04	0.13 ± 0.01
Uterus	0.79 ± 0.44	0.54 ± 0.22	0.57 ± 0.23	0.58 ± 0.14	0.56 ± 0.20	0.66 ± 0.11

### 3.4 Mutagenicity studies

#### 3.4.1 Bacterial reverse mutation test (Ames assay)

Q-Actin did not cause an increase in the number of revertant colonies in *S. typhimurium* strains TA98, TA100, TA102, TA1535 and TA1537 and no precipitation was observed at the tested concentrations (50, 150, 500, 1500 and 5000 μg/plate) in the presence and absence of rat metabolic activation system in either preincubation or plate incorporation method (data not shown). The study was considered as acceptable since the positive control produced a significant increase in mutant colony frequencies and the spontaneous reversion rates in the negative control were within three times the standard deviation of the means of normal range and not more than 5% of plates were lost through contamination.

#### 3.4.2 *In vitro* chromosomal aberration assay

There was no cytotoxicity observed at Q-Actin concentration of 0.20, 0.41, 0.83, 1.65 and 3.33 mg/ml both with and without metabolic activation (data not shown). In tests concentrations (0.83, 1.67, 3.33 mg/ml) of Q-Actin, a chromosomal break was observed at 3.33 mg/ml concentration in the long-term test without metabolic activation. There was no statistical difference in the revertant count in any of the Q-Actin test concentrations with or without metabolic activation compared to the vehicle controls (data not shown). In the chromosome aberration test, negative controls both with and without metabolic activation had no aberrations while the percentage of aberrations in the positive control with S-9 activation short term test showed a 42% incidence of aberration (28 chromatid gaps, 26 chromosomal gaps, 31 chromatid breaks and 38 chromosomal breaks). The positive controls in the short- and long-term tests without S-9 activation showed 40.33% (33 chromatid gaps, 35 chromosomal gaps, 21 chromatid breaks and 32 chromosomal breaks) and 39.33% (20 chromatid gaps, 27 chromosomal gaps, 32 chromatid breaks and 40 chromosomal breaks) aberrations (data not shown).

#### 3.4.3 *In vivo* micronucleus test

There were no statistically significant increases in the frequency of micronuclei at any dose level after administration of Q-Actin (250, 500 or 1000 mg/kg bw) to mice as compared to the vehicle control. Positive controls showed statistically significant increase of induced micronucleus frequency (Table 6).

**Table 6:** Summary of micronucleus test results.

Group (n = 10)	Dose (mg/kg bw)	Sampling time (hr)	PCE with micronuclei	PCE per 500 erythrocytes (mean ± SD)
G1 (Vehicle control)	0	24	0.12	250 ± 1.5
G2 (Q-Actin low dose)	250	24	0.10	252.5 ± 4.9
G3 (Q-Actin mid dose)	500	24	0.10	250.4 ± 2.5
G4 (Q-Actin high dose)	1000	24	0.11	251.2 ± 4.6
G5 (Q-Actin high dose)	1000	48	0.10	250.9 ± 3.4
G6 (Cyclophosphamide)	30	24	0.64*	298.4 ± 14.6*
G7 (Methyl methanesulfonate)	50	24	0.42*	334.3 ± 11.7*

* p < 0.01 compared to G1.

## 4 Discussion

In recent years, the discovery and identification of iminosugars in commonly consumed fruits and vegetables has led to an increased interest in the benefits of these compounds on human health. Recent investigations have explored the potential biological activities of several similar iminosugars, including 1-Deoxynojirimycin (DNJ) from mulberry and ido-BR1 from cucumber. Prichard et al. [21] reviewed several iminosugar derivatives with reported activities including glycosidase inhibition, and immunosuppressant, antibacterial, antiviral and anti-inflammatory effects. The iminosugar found in mulberry, DNJ, has garnered interest for antihyperglycemic, anti-obesity, and anti-viral biological activities [9]. The biological activities of iminosugar does not indicate potential for toxicity. A 28-day repeated dose toxicity study in rats using a commercially available mulberry leaf extract standardized to 5% DNJ, found no safety concerns [22]. This study concluded that the no observed adverse effect level was 4000 mg/kg bw/day (the highest dose tested), supporting the safety of DNJ and similar iminosugars.

Iminosugars inhibit the glycosidases involved in a wide range of important biological processes because of their structural resemblance to the sugar moiety of the natural substrate and the presence of the nitrogen atom mimicking the positive charge of the glycosyl cation intermediate in the enzyme-catalyzed glycoside hydrolysis. These iminosugars and their derivatives have received considerable attention for their potential uses. Cucumber fruit contains a unique iminosugar, ido-BR1, shown to have anti-inflammatory properties, prompting interest in human application. In a six-month (180 days) randomized, double-blind clinical trial in 122 patients diagnosed with osteoarthritis, Nash & Azantsa [12] compared the effects of cucumber extract (Q-Actin) with that of a combination of glucosamine hydrochloride and chondroitin sulphate (GC). The patients received either 10 mg of Q-Actin or 1350 mg of GC (control) twice a day for 180 days. In addition to efficacy parameters, adverse events were recorded and clinical laboratory tests such as hematology, clinical chemistry and urinalysis were conducted initially and at the end of the study (180 days). No adverse events were reported during the study and the clinical laboratory tests did not reveal any treatment related changes or adverse effects.

Q-Actin, a novel aqueous extract of cucumber standardized to ≥1% ido-BR1 iminosugar was studied to confirm its safety in use. The results of mutagenicity studies of Q-Actin, as evaluated by bacterial reverse mutation test (Ames assay), *in vitro* chromosomal aberration assay and *in vivo* micronucleus test in Swiss albino mice, did not reveal any genotoxic effects. In an earlier study, Stoltz et al. [23] reported that an extract of cucumber in an Ames test using *S. typhimurium* TA98 and TA100 with and without metabolic activation was not mutagenic. The findings from the present study support these observations. The results of the present dose-response repeat-dose animal toxicity study showed that oral (gavage) administration of Q-Actin at levels up to 1000 mg/kg bw/day (the highest dose tested) to male and female Sprague Dawley rats for up to 90-days was not associated with adverse effects as evaluated by the general condition and appearance of the animals, growth, feed consumption, ophthalmoscopy, hematology, clinical chemistry, urinalysis or organ weights, necropsy, and histopathological examination of organs/tissues. A few statistically significant differences between the control and treatment groups were noted in the 90-day study but none were regarded to represent adverse effects of Q-Actin as described below. These findings suggest that daily administration of Q-Actin to rats at doses up to 1000 mg/kg bw/day (the highest dose tested) for 90-days was well tolerated. The findings of this study are supported by a previous 28-day rat toxicity study in which mulberry leaf extract containing iminosugar (5% DNJ) did not reveal any adverse effects and the NOAEL was found to be 4000 mg/kg bw/day [22].

In the present study, statistically significant differences in body weight and body weight gain observed in male and female groups during few isolated time periods were not considered to be toxicologically important because there was no dose-response effect and there were no significant differences at the end of the study period. Significant increases in absolute organ weights of the kidney, spleen and thymus and decreases in the relative organ weights of the adrenal glands and thymus in the G4R (high dose) female group were considered as unrelated to treatment as there were no dose-response effect, and no correlated findings in the histopathology. Hematological parameters showed an isolated incidence of statistically significant increase in lymphocytes (LYM) in G2 females. As the increase in LYM was associated with only one sex, and did not show a dose-response effect, the finding was considered incidental and not related to Q-Actin. Clinical chemistry results showed a statistically significant decrease of in G3 and G4 females (Table 4). These changes were not toxicologically relevant as the changes were of low magnitude and changes were recovered at the end of the recovery period.

In summary, the findings from present mutagenicity and animal toxicity studies suggest that Q-Actin aqueous cucumber extract is unlikely to cause any genotoxic effects or adverse effects in animals. The results of present subchronic toxicity studies suggest that oral administration of Q-Actin at levels up to 1000 mg/kg bw/day does not cause adverse effects in male and female rats. Based on the results of the 90-day study, the no-observed-effect-level (NOAEL) of Q-Actin aqueous cucumber extract was found to be more than 1000 mg/kg bw/day, the highest dose tested. For a human subject weighing 60 kg the equivalent cucumber extract (Q-Actin) dose from the animal study where no adverse effects were noted will be 60,000 mg/person/day. The findings from the present subchronic animal toxicity study, where a 6000-fold higher dose as compared to the human study (10 mg/day) in which no adverse effects were observed (Nash & Azantsa, [12], further support the safe use of Q-Actin in human.

## Conflict of interest

M. Saravanan and S. Muthusamy are employees of Vipragen Biosciences Pvt. Ltd, where the studies were performed. S. Kothari is employed by Gateway Health Alliances. A. Mozingo works as an independent consultant and M. Soni works as an independent consulting toxicologist.

## Transparency document

Transparency Document
